# Complete Chloroplast Genome Sequence of *Sonchus brachyotus* Helps to Elucidate Evolutionary Relationships with Related Species of Asteraceae

**DOI:** 10.1155/2021/9410496

**Published:** 2021-12-01

**Authors:** Caixiang Wang, Juanjuan Liu, Yue Su, Meili Li, Xiaoyu Xie, Junji Su

**Affiliations:** College of Life Science and Technology, Gansu Agricultural University, Lanzhou 730070, China

## Abstract

*Sonchus brachyotus* DC. possesses both edible and medicinal properties and is widely distributed throughout China. In this study, the complete cp genome of *S. brachyotus* was sequenced and assembled. The total length of the complete *S. brachyotu*s cp genome was 151,977 bp, including an LSC region of 84,553 bp, SSC region of 18,138 bp, and IR region of 24,643 bp. Sequence analyses revealed that the cp genome encoded 132 genes, including 87 protein-coding genes, 37 tRNA genes, and 8 rRNA genes. The GC content was 37.6%. One hundred mononucleotide microsatellites, 4 dinucleotide microsatellites, 67 trinucleotide microsatellites, 4 tetranucleotide microsatellites, and 1 long repeat were identified. The SSR frequency of the LSC region was significantly greater than that of the IR and SSC regions. In total, 175 SSRs and highly variable regions were recognized as potential cp markers. By analyzing the IR/LSC and IR/SSC boundaries, structural differences between *S*. *brachyotus* and 6 other species were detected. According to phylogenetic analyses, *S. brachyotus* was most closely related to *S*. *arvensis* and *S. oleraceus*. Overall, this study provides complete cp genome resources for *S. brachyotus* that will be beneficial for identifying potential molecular markers and evolutionary patterns of *S. brachyotus* and its closely related species.

## 1. Introduction

*Sonchus* L. is a genus of annual, biennial, or perennial herbaceous plants in the Asteraceae (Compositae) family. Currently, the *Sonchus* genus includes 95 species [1–3], and it is widely distributed throughout Europe, Asia, Africa, and Pacific Islands [2]. Only 8 of the 95 species are distributed throughout China: *Sonchus arvensis*, *Sonchus asper*, *Sonchus brachyotus*, *Sonchus lingianus*, *Sonchus oleraceus*, *Sonchus palustris*, *Sonchus transcaspicus*, and *Sonchus uliginosus*. Specifically, they are distributed in Northeast, Northwest, North, Central, and South China and other regions and grow on mountain grassy slopes, roadsides, and fields with very rich resources, according to the Flora of China [4].

In China, *S. brachyotus* can be used not only as food but also as medicine to treat diseases [5]. *S. brachyotus* contains many major and trace elements that are important for the health and metabolism of the human body and is often used in health products as an edible plant [6–8]. When *S. brachyotus* is used as a medicinal whole herb, it has the function of clearing heat, detoxification, cooling blood, and stopping bleeding, and it is often used in the treatment of diseases such as acute pharyngitis, acute dysentery, appendicitis, enteritis, and hemorrhoids [6, 9]. A previous study showed that *S. brachyotus* has antimicrobial activities against several pathogenic microorganisms [8]. For example, an extract from *S. brachyotus* can induce the apoptosis of A549 cells and inhibit their growth and proliferation, indicating that *S. brachyotus* can potentially be used to prevent and restrain tumor growth [10]. Pan et al. [11] also showed that an extract from *S. brachyotus* could exhibit antimicrobial activity against *Escherichia coli*, *Enterobacter cloacae*, *Klebsiella pneumoniae*, *Salmonella enterica*, *Staphylococcus aureus*, and *Micrococcus luteus*; this is especially true in the case of *Escherichia coli.* In addition, functional antioxidant components of *S. brachyotus*, including caffeic acid, rutin, orientin, and luteolin, can scavenge free radicals [12]. Although the chemical composition of *S. brachyotus* has been reported, *S. brachyotus* and *S. arvensis* are similar in morphology and difficult to distinguish, and their phylogenetic relationships are not very clear.

The chloroplast is an important plastid that provides necessary energy for growth via photosynthesis and plays vital roles in the physiology and development of plants. Chloroplasts, as semiautonomous organelles, possess a genetic information expression system. In contrast to nuclear DNA, chloroplast (cp) DNA exhibits single-parent inheritance. The cp genome is more conserved than mitochondrial and nuclear genomes in terms of gene type, genome organization, and genome structure [13], so the cp genome has become an important means for reconstructing the phylogenetic relationships among plant species [14–21]. With the development of bioinformatics analysis and sequencing technology, studies on the evolution of species using cp genome sequences are increasing.

In this study, we sequenced and analyzed the complete cp genome of *S*. *brachyotus* and reconstructed the phylogeny of Compositae based on the cp genomes of 42 species. The following questions were addressed: (1) what are the features of the cp genome of *S*. *brachyotus*? (2) How many potential microsatellite markers can the cp genome provide? (3) Which types of structural variation events have occurred across the cp genomes in the *Sonchus* genus?

## 2. Materials and Methods

### 2.1. Preparation of Materials

The plant material was transplanted from the shore of the Yellow River in Anning District of Lanzhou, Gansu Province, China (36°5′10′N, 103°34′47′E) to pots in a laboratory (Figure S1). Then, fresh leaf tissue (1–2 grams) was sent to Genepioneer Biotechnologies Inc., Nanjing, 210023, China, for sequencing.

### 2.2. DNA Extraction, Genome Sequencing, and Annotation

Total genomic DNA was extracted from 100 mg of fresh leaves of *S. brachyotus* using the CTAB (cetrimonium bromide) method [22]. The Illumina NovaSeq 6000 platform was used to construct and sequence a genomic library on the basis of the standard Illumina paired-end (PE) protocol. The raw reads were trimmed using NGS QC Toolkit_v2.3.3 [23]. After trimming of low-quality reads and adapter sequences, the clean reads were aligned with the reference genome of *Lactuca sativa* (NC_007578.1) and *S. arvensis* (NC_054161) from the NCBI GenBank database using Burrows-Wheeler Alignment (BWA) [24], and sequenced reads of chloroplast genomes were “selected” from clean sequence data. The matched PE reads were assembled using SPAdes v3.10.1 software [25]. The reference sequences of the genomes were compared for collinearity of conserved and rearranged genomes by MUMmer v3.23 [26]. Annotation was performed with BLAST v2.2.25 (https://blast.ncbi.nlm.nih.gov/Blast.cgi), HMMER v3.1b2 (http://http://www.hmmer.org/), and Aragorn v1.2.38 (http://130.235.244.92/ARAGORN/). BLAST v2.2.25 was applied to compare coding sequences (CDSs) of chloroplasts in the NCBI database, the preliminary draft annotation was examined and adjusted manually by comparison with the reference cp genome, and the gene annotation results of the cp genome were then obtained. The rRNA and tRNA annotation information was obtained by using HMMER v3.1b2 and Aragorn v1.2.38 to compare the rRNA and tRNA sequences of chloroplasts in the NCBI online database. The annotated cp DNA sequences were submitted to the NCBI database by BankIt to obtain the GenBank sequence login number MT850048. OGDRAW v1.1.1 software [27] was then used to map the cp genomes of *S. brachyotus* according to the chloroplast sequence assembly results.

### 2.3. Repeat Structure and Sequence Analysis

Vmatch v2.3.0 (http://www.vmatch.de/) was utilized to explore the reduplicative structure of the cp genome of *S. brachyotus* and to locate a variety of styles of repeat sequences for forward, palindromic, inverted, and complementary sequences. The parameters were set to a minimum length of 30 bp and a Hamming distance of 3. Microsatellite (mono-, di-, tri-, tetra-, penta-, and hexanucleotide repeats) detection was performed using MISA v1.0 (http://pgrc.ipk-gatersleben.de/misa/misa.html), with parameters of 1–8 (mononucleotide motifs with a minimum of 8 repetitions), 2–5, 3–3, 4–3, 5–3, and 6–3.

### 2.4. Identification of Highly Divergent Regions

We used published cp genome sequences of 6 species of *Sonchus*, namely, *Sonchus webbii* (GenBank accession number NC_042383), *Sonchus acaulis* (NC_042382), *Sonchus canariensis* (NC_042381), *Sonchus boulosii* (NC_042244), *Sonchus arvensis* (NC_054161.1), and *Sonchus oleraceus* (MG878405), to analyze the borders and synteny of the inverted repeat (IR) and single-copy (SC) regions of *S. brachyotus* and the above 6 species. We used IRScope software (https://irScope.shinyapps.io/Irapp/) to generate a comparison diagram of the IR boundary [28]. Entire genome sequences were evaluated to appraise realignments and extensive sequence variances using Mauve 2.3.1 [29]. Moreover, the cp genome was arranged using MAFFT v7.427 [30] to identify divergence hotspots, after which sliding window analyses were conducted via DnaSP v5 [31] to determine the nucleotide diversity (Pi) of the complete cp.

### 2.5. Phylogenetic Analyses

A total of 43 cp genomes available in GenBank were recovered to infer the phylogenetic relationships, including newly sequenced *S. brachyotus* and 42 published Compositae species (Table S1). Multiple alignments were performed using complete cp genomes based on the conserved structure and gene order of the chloroplast genomes. All the nucleotide sequences were aligned using MAFFT v7.308 [32] to assess the taxonomic and phylogenetic relationships of *S. brachyotus*. Two methods were employed to construct phylogenetic trees, including maximum parsimony (MP) and Bayesian inference (BI). MP analyses were performed using Mega 11.0 software [33], and the addition sequence was set as 1,000 replications for the heuristic search. BI analyses were conducted using MrBayes v3.2.6 [34] based on the model GTR+G inferred from Modeltest 3.7 [35]. The first 25% of trees generated were discarded as burn-in, and the remaining trees were used to construct a majority-rule consensus tree with posterior probability (PP) values for each node.

## 3. Results

### 3.1. Chloroplast Genome Features, Sequencing, and Assembly of *S. brachyotus*

**Figure 1:**
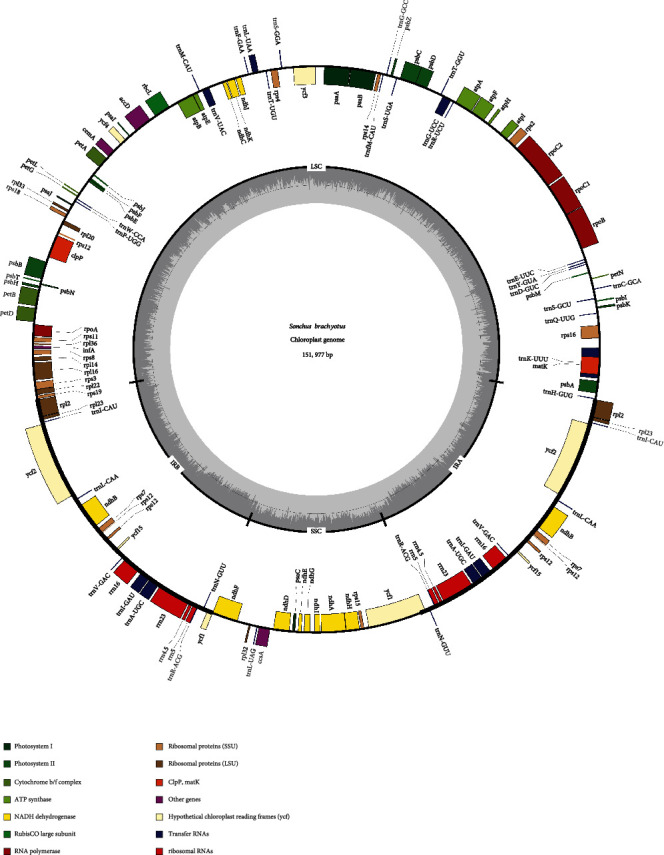
Gene map of the *S. brachyotus* chloroplast genome. The genes inside and outside of the circle are transcribed in the clockwise and counterclockwise directions, respectively. Genes belonging to different functional groups are indicated in different colors. The thick lines indicate the extent of the inverted repeats (IRa and IRb) that separate the genomes into small single-copy (SSC) and large single-copy (LSC) regions.

After trimming of low-quality reads and adapter sequences, the total length of the reads was approximately 7.5 Gb and 24,858,121 clean reads were produced by the Illumina NovaSeq 6000 platform. Based on a combination of de novo and reference-guided assembly, the cp genome of *S. brachyotus* was obtained. The complete cp genome sequence of *S. brachyotus* was submitted to the NCBI database under GenBank accession number MT850048. The total length of the cp genome of *S. brachyotus* was 151,977 bp (Table 1, Figure 1). The cp genome contained four characteristic regions: a large single-copy (LSC) region of 84,553 bp, a small single-copy (SSC) region of 18,138 bp, and a pair of inverted repeats (IRa and IRb) of 24,643 bp. The base composition of the complete cp genome sequence was analyzed and found to be 31.3% T, 31.1% A, 18.7% C, and 18.9% G. The overall GC content was 37.6%, which is very close to those of other *Sonchus* species. Furthermore, the GC contents were unevenly distributed across regions of the cp genome and were found to be 35.71%, 31.44%, and 43.08% for the LSC, SSC, and IR regions, respectively.

The *S. brachyotus* cp genome included 132 genes, 1 or 2 more genes than the other 6 *Sonchus* genomes, of which there were 87 protein-coding genes, 8 rRNA genes, and 37 tRNA genes (Table 1). Eight protein-coding genes (*ndhB*, *rpI2*, *rpI23*, *rps7*, *rps12*, *ycf2*, *ycf15*, and *ycf1*), 7 tRNA genes (*trnI-CAU*, *trnL-CAA*, *trnV-GAC*, *trnI-GAU*, *trnA-UGC*, *trnR-ACG*, and *trnN-GUU*), and 4 rRNA genes (*rrn16*, *rrn23*, *rrn4.5*, and *rrn5*) were duplicated in the IR region in the cp genomes. There were 113 unique genes, and 16 genes (*trnK-UUU*, *rps16*, *rpoC1*, *atpF*, *trnG-UCC*, *trnL-UAA*, *trnV-UAC*, *rps12*, *petB*, *petD*, *rpl16*, *rpl2*, *ndhB*, *trnI-GAU*, *trnA-UGC*, and *ndhA*) contained 1 intron, whereas 2 protein-coding genes (*ycf3* and *clpP*) contained 2 introns (Table 2). The majority of these intron-containing genes were located in the LSC region.

### 3.2. Simple Sequence Repeats and Large Repeat Sequences

**Figure 2:**
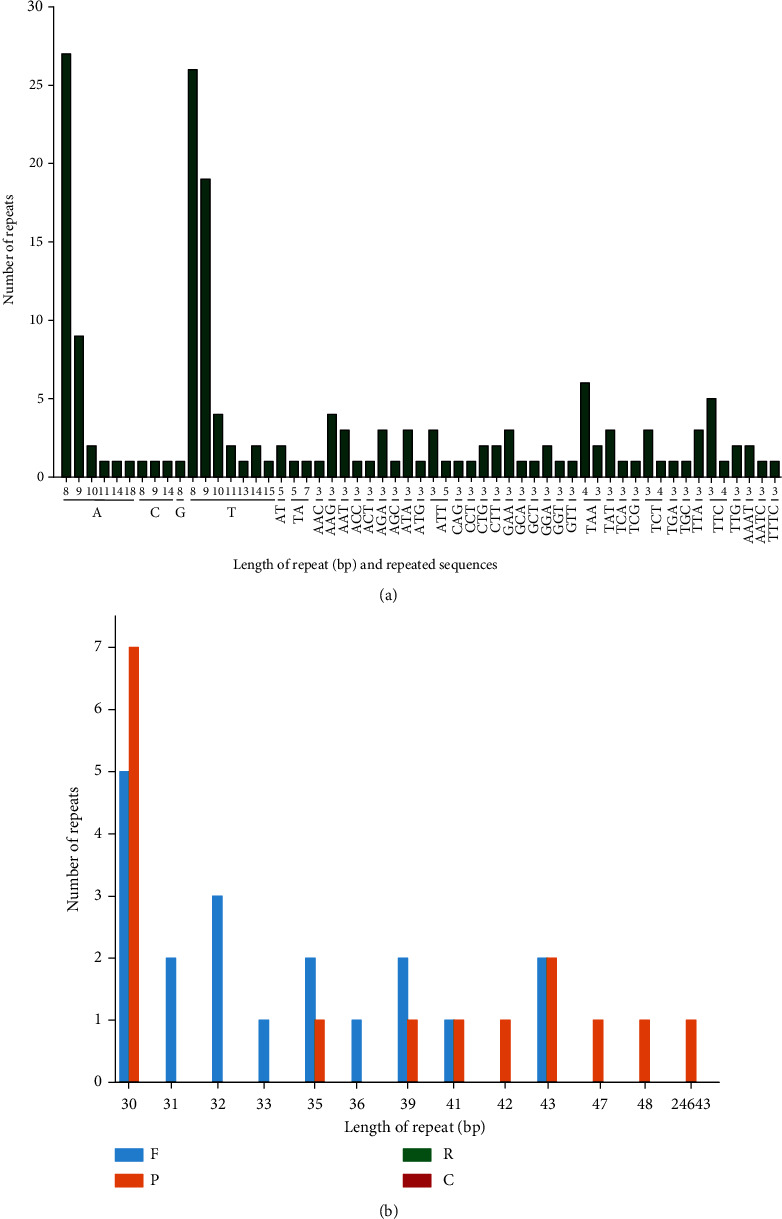
SSR numbers according to their distribution and repeat type and repeat numbers according to repeat type and repeat length in *S. brachyotus*. (a) Number of SSR motifs in *S. brachyotus*. (b) Variation in the distribution of forward (F), reverse (R), complementary (C), and palindromic (P) repeats and the number of different repeats in the chloroplast genome of *S. brachyotus*.

In this study, we explored the presence of various microsatellites (mono-, di-, tri-, tetra-, penta-, and hexanucleotides) in the cp genome of *S. brachyotus*. A total of 175 microsatellites were detected in the cp genome of *S. brachyotus*, and the most common simple sequence repeats (SSRs) were mononucleotides (notably for A/T), with 100, accounting for 57% of the SSRs in *S. brachyotus*. The second most abundant motif type was the trinucleotide type, especially TAA, with a total number of 67 in *S. brachyotus* (approximately 38%). The proportion of other SSR types was relatively low (approximately 2% for dinucleotides and tetranucleotides). Intriguingly, the SSRs in *S. brachyotus* were chiefly distributed in coding regions (46.5%), with much lower numbers distributed in noncoding introns (12.6%) and intergenic regions (41%). The SSRs were spaced disproportionately through the cp genome, with the largest number of SSRs situated in the LSC region, followed by the IR and SSC regions, in the quadripartite structure regions (Figure 2(a)).

Repeat motifs are valuable for phylogenetic reconstruction. Consequently, we examined the forward, palindromic, complementary, and reverse repeats in the *S. brachyotus* cp genome (Figure 2(b)). Overall, 35 pairs of repeat sequences were identified in the cp genome of *S. brachyotus*, which contained 16 palindromic repeats and 19 forward repeats; however, complementary and reverse repeats were not found in *S. brachyotus*. The lengths of the repeats ranged from 30 to 24,643 bp in *S. brachyotus*, and the most common repeat length was 30 bp (approximately 34%), followed by repeats of 43 bp (11%) and 31–42 bp (approximately less than 10%), while those of 43–24,643 bp (approximately 2%) were comparatively rare. The repeats were mainly distributed in noncoding regions, including intergenic spacers (IGSs) and introns. However, several coding and tRNA genes, such as *ycf2*, *ycf3*, *psbN*, *psaB*, *psaA*, *ndhA*, *rpI16*, and *trnS*, also contained repeat sequences.

### 3.3. Expansion and Contraction of Border Regions

**Figure 3:**
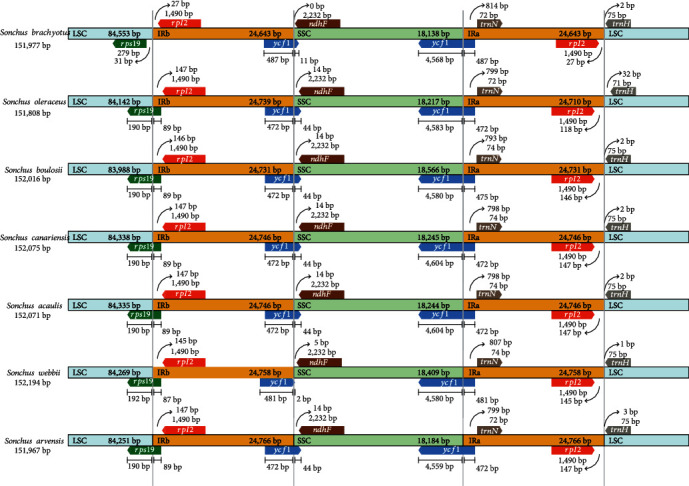
Comparison of the border positions of LSC, SSC, and IR regions among 7 chloroplast genomes from *Sonchus* species. Gene names are indicated in boxes.

The expansion and contraction of the borders and adjacent genes of cp genomes give rise to genome size variations among various plant lineages. Hence, the borders and adjacent genes of the other 6 published *Sonchus* plant species were compared with those of *S. brachyotus* to analyze the expansion and contraction diversification in connection regions (Figure 3). The entire genome structure, the gene order, and the gene number were conserved, as were the IRb/SSC and IRa/LSC boundaries of the seven *Sonchus* cp genomes. The *rps19* genes in the LSC region of the 6 species were amplified and generated products of 87 and 89 bp (89 bp for *S. oleraceus*, *S. boulosii*, *S. canariensis*, *S. acaulis*, and *S. arvensis*; 87 bp for *S. webbii*) for the IRb region; in *S. brachyotus*, this gene was completely situated in the LSC region, and the distance to the connection was 31 bp. The *rpl2* gene in the IR regions was 27, 145, 146, and 147 bp from the LSC in the 7 species (27 bp for *S. brachyotus*; 145 bp for *S. webbii*; 146 bp for *S. boulosii*; and 147 bp for *S. oleraceus*, *S. canariensis*, *S. acaulis*, and *S. arvensis*). The *trnH* gene in the LSC region was contracted by 1, 2, 3, and 32 bp from the connection region of IRa/LSC (1 bp for *S. webbii*; 2 bp for *S. brachyotus*, *S. boulosii*, *S. canariensis*, and *S. acaulis*; 3 bp for *S. arvensis*; and 32 bp for *S. oleraceus*). The *ycf1* gene spanning the SSC/IRb junction showed a length of 44 bp in *S. oleraceus*, *S. boulosii*, *S. canariensis*, *S. acaulis*, and *S. arvensis*, but in *S. brachyotus* and *S. webbii*, it showed a length of 11 and 2 bp. The *ndhF* gene was located completely within the SSC region, and the distance to the IRb/SSC junction was 0, 5, and 14 bp. The *ycf1* gene extended over the boundary region between the SSC and IRa regions. The *trnN* gene was located entirely within IRa and was contracted by 793–814 bp. The variations in the IR/SC boundary regions in the 7 *Sonchus* cp genomes were responsible for the length differences in the four regions and whole genome sequences.

### 3.4. Sequence Divergence and Hot Spots

**Figure 4:**
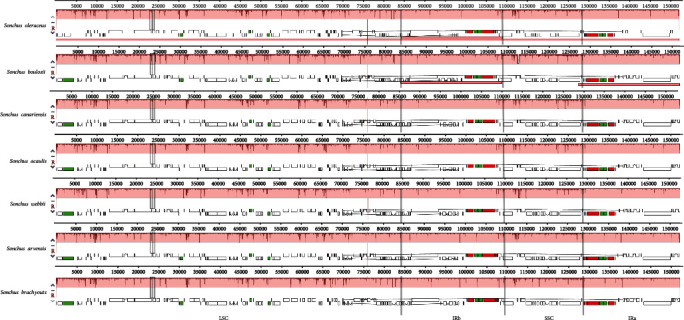
Mauve alignment of the 7 *Sonchus* chloroplast genomes. The rectangles represent the similarity between genomes, and the lines between rectangles represent a type of collinearity. The small square indicates the gene location in each genome. White represents CDSs, green represents tRNAs, and red represents rRNAs.

To clarify the level of genomic differences, the cp genome sequences of *S. brachyotus* plants were compared via Mauve. The local collinear block sequences (LCBSs) confirmed by Mauve showed high sequence similarity among the 7 *Sonchus* cp genomes, which indicated that the genome structure was quite conserved at the gene sequence level (Figure 4). As anticipated, the SC regions were less conserved than the IR regions. The most divergent areas were 5,000–20,000, 25,000–40,000, 45,000–80,000, and 110,000–130,000 bp in size.

**Figure 5:**
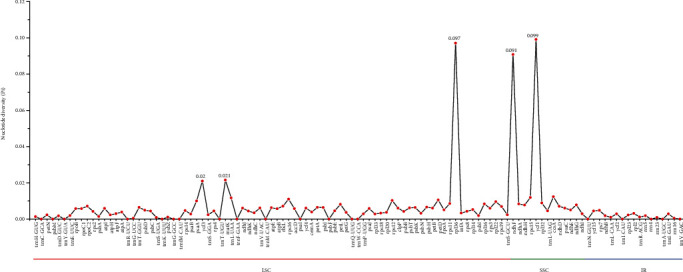
Nucleotide diversity (Pi) values among the 7 *Sonchus* species.

We generated 113 loci from *S. brachyotus* and calculated the Pi value of each gene with VCFtools. The Pi values obtained from *S. brachyotus* ranged from 0 to 0.099 (*ycf1*). The number of variable sites in the IR region was more conserved than that in the LSC and SSC regions, and 5 of these sites were highly variable: *ycf3*, *matK*, *rpl36*, *ndhF*, and *ycf1*. Three of the sites (*ycf3*, *matK*, and *rpl36*) were located in the LSC region, and 2 (*ndhF* and *ycf1*) were located in the SSC region (Figure 5). Five divergence hotspots in the most variable regions (Pi > 0.02) could be used as potential molecular markers for phylogenetic studies of *Sonchus* species.

### 3.5. Phylogenetic Analysis

**Figure 6:**
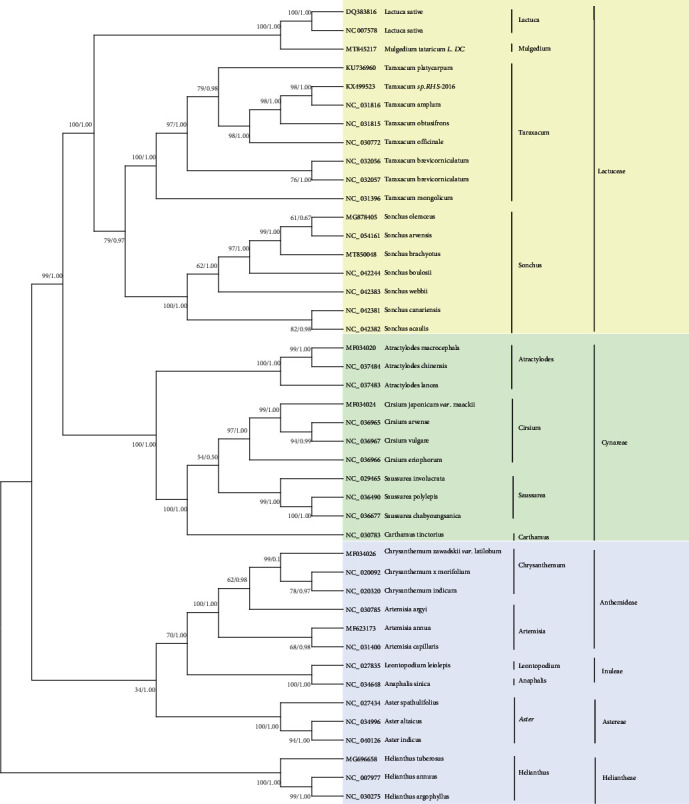
Phylogenetic analysis of the chloroplast genomes of 43 Compositae plants using maximum parsimony and Bayesian inference. MP bootstrap value/Bayesian posterior probability given at each node. The pentastar in the phylogenetic tree indicated that the support rate of the branch is 100/1.0. The yellow area is the first branch, the green area is the second branch, and the purple area is the third branch.

On the basis of the phylogenetic analysis of the cp genome relationships of 42 representative Compositae plants, the taxonomic status and evolutionary relationships of *S. brachyotus* were determined (Figure 6). The evolutionary tree revealed clear phylogenetic relationships for 43 species in 14 genera of Compositae, which were clustered into 3 branches. The first branch consists of 18 species in 4 genera, *Lactuca*, *Mulgedium*, *Taraxacum*, and *Sonchus*, all belonging to Lactuceae. The second branch consists of 11 species from 4 genera, *Atractylodes*, *Cirsium*, *Carthamus*, and *Saussurea*. The third branch consists of 14 species of 6 genera, *Chrysanthemum*, *Artemisia*, *Leontopodium*, *Aster*, *Anaphalis*, and *Helianthus*. *Chrysanthemum* and *Artemisia* belong to Anthemideae; *Leontopodium* and *Anaphalis* belong to Inuleae; *Aster* belongs to Astereae; and *Helianthus* belongs to the Heliantheae. These are all members of Cynareae. *Sonchus* is located on the first branch of the phylogenetic tree. In the *Sonchus* genus, *S. brachyotus* is more closely related to the small clades formed by *S. arvensis* and *S. oleraceus*, so it can be inferred that they have the closest relationship.

## 4. Discussion

As the second largest family in the plant kingdom, Compositae consists of approximately 1,620 genera and more than 23,600 species [36, 37]. Nevertheless, few cp genomic sequences for members of this family have been stored in GenBank, with the first sequence being that of *L*. *sativa* [38, 39]. Although the advancement of high-throughput sequencing techniques has enabled several additional Compositae cp genomes to be sequenced [40–43], the cp genome of *S*. *brachyotus* has remained unexplored. In this study, we sequenced the complete cp genome of *S. brachyotus* by using Illumina high-throughput sequencing technology.

The structure and genes of the cp genome of *S. brachyotus* were found to be highly conserved through comparative analysis with closely related species, and they exhibited the same protein-coding genes, tRNAs, and rRNAs. Nevertheless, there was a difference in genome size (Table 1), indicating genetic differences. We found that this phenomenon may be due to contractions and expansions of boundary regions [44–48]. The length of the cp genome sequence is related to the contraction and expansion of noncoding regions. Recent studies have revealed that the IRb/SSC and IRa/LSC regions are mainly responsible for length differences in cp genome sequences, and such regions have been discovered in numerous angiosperm cp genome sequences [49]. Cho et al. [1, 50] carried out a boundary analysis of the LSC, SSC, and IR regions of the cp genomes of 5 *Sonchus* plants and found some slight differences in the position or length of the *rps19*, *rpl2*, *trnH*, *ndhF*, and *ycf1* genes. Although the whole genome structure, including both gene number and order, was found to be nearly identical, the cp genome of *S. brachyotus* and the 6 published cp genomes of *Sonchus* (*S*. *oleraceus*, *S*. *boulosii*, *S*. *canariensis*, *S*. *acaulis*, *S*. *webbii*, and *S*. *arvensis*) showed obvious deviations at the IRb/SSC and IRa/LSC borders.

Microsatellites can be divided into mono-, di-, tri-, tetra-, penta-, and hexanucleotide repeats. The locations of SSRs have functional roles in the genome, including gene regulation, advancement, and evolution. As shown in a genome-wide analysis of polymorphisms related to height, microsatellite markers can be powerful tools for measuring genetic diversity in populations and addressing genetic issues, such as gene origin, flow, and species group configuration, at the level of both intraspecific and interspecific variations [51]. Population-specific cp SSR polymorphisms have also been documented in other plant species, such as *Pinus sylvestris* L. [52], *Triticum* spp. [53], *Abies alba* Mill. [54], and *Cucumis* spp. [55]. Repeat motifs play a crucial role in phylogenies, and they are valuable because of their applicability to genome rearrangement analysis [56]. Cho et al. [1, 50] analyzed the SSRs of the cp genomes of 5 species of *Sonchus* and found that the SSRs were mainly distributed in coding regions and LSC regions. In our study, 175 repeat sequences were found in *S. brachyotus*; additionally, we discovered that they mostly existed in the LSC regions.

Previous studies show that multiple sequence alignments used for interspecies discrimination can reveal the development of mutational hotspots [57, 58] and be applied in phylogenetic or phylogeographic studies [59, 60]. At present, some studies have shown that markers derived from chloroplast genomes can also be used in phylogenetic studies [61]. In several studies, the LSC and SSC regions were less conserved than the IR region [61–63], as revealed in this study. Numerous variable sites (e.g., *ycf3*, *matK*, *rpl36*, *ndhF*, and *ycf1*) were confirmed by calculating and comparing the nucleotide diversity value (Pi). Among them, *ycf1* and *ycf3* have been demonstrated to be conducive markers for phylogenetic studies of *Sonchus* [1, 50]. These markers were also found to be useful for analyzing the intraspecific variation of *S. brachyotus*. According to the results of the present study, 5 divergence hotspots screened on the basis of Pi > 0.02 show great potential for the development of a system of highly informative markers for *S. brachyotus*.

The taxonomic position and evolutionary relationships of *S. brachyotus* were revealed through comparisons with 42 Compositae plants, which were based on the correlations of all cp genomes. The 43 Compositae plants were divided into 3 groups. The phylogenetic relationships identified among *Sonchus* species were consistent with those from previous studies [1, 50, 64]. James et al. [64] constructed a phylogeny of 13 species of Compositae plants on the basis of the cp genome and revealed that *S*. *oleraceus* was closely related to *L*. *sativa* (AP007232). Cho et al. [1] used cp genomes to analyze a phylogeny of 32 Compositae plants and revealed that *S*. *acaulis*, *S*. *canariensis*, and *S*. *webbii* were closely related to *S*. *oleraceus* (MG 878405). Cho et al. [50] utilized cp genomes to analyze a phylogeny of 30 Compositae plants and demonstrated that 2 *S. asper* and 2 *S*. *oleraceus* plants were closely related to *S*. *oleraceus* (MG 878405). Overall, *S*. *oleraceus* was closely related to *S*. *asper*. In this study, *Sonchus* was most closely related to *Taraxacum*, followed by *Lactuca*. *S*. *arvensis* is the closest relative of *S*. *brachyotus*, followed by *S*. *oleraceus*, within the *Sonchus* genus. Therefore, we hypothesize that *S. brachyotus* and *S*. *arvensis* show similarity in physiology. Phylogenetic relationships identified within *Sonchus* and its phylogenetic relationships with other genera of the Compositae can facilitate additional studies. The cp genome sequences provide useful genetic information for understanding the evolution of Compositae plants.

## 5. Conclusions

In this study, we assembled, annotated, and analyzed the cp genome of *S. brachyotus*, an important wild plant used for food and medicine. The *S. brachyotus* cp genome (151,977 bp) was fully characterized and compared with those of related species. We identified IR regions, as well as SSC and LSC regions. The *S. brachyotus* cp genome included 132 genes, of which there were 87 protein-coding genes, 8 rRNA genes, and 37 tRNA genes. A total of 175 microsatellites and 35 pairs of repeat sequences were detected in the cp genome of *S. brachyotus*. The unique inversion, insertion, and gene loss events detected here may provide informative markers for phylogenetic resolution among different genera in Compositae. Several hotspots (e.g., *ycf3*, *matK*, *rpl36*, *ndhF*, *and ycf1*) of intergeneric divergence were also identified. Both RAxML and GTR analyses strongly support the topology in which the clade including *S*. *brachyotus* is near that containing *S*. *arvensis*. The cp genomic resources presented in this study will be useful for further studies on the evolutionary patterns of *S. brachyotus* and its closely related species.

## Figures and Tables

**Table 1:** Summary of the features of 7 *Sonchus* chloroplast genomes.

Taxon	Length (bp)				Number of genes				GC content (%)
	Genome	LSC	SSC	IR	Total	Protein coding	tRNA	rRNA	
*S*. *brachyotus*	151,977	84,553	18,138	24,643	132	87	37	8	37.6
*S*. *arvensis*	151,967	84,251	18,184	24,766	130	87	37	6	37.6
*S*. *oleraceus*	151,808	84,142	18,217	24,739	130	87	37	6	37.6
*S*. *boulosii*	152,016	83,988	18,566	24,731	130	88	36	6	37.6
*S*. *acaulis*	152,017	84,355	18,244	24,746	131	88	37	6	37.6
*S*. *canariensis*	152,075	84,338	18,245	24,746	131	88	37	6	37.6
*S. webbii*	152,194	84,269	18,409	24,758	131	88	37	6	37.6

LSC: large single copy; SSC: small single copy; IR: inverted repeat; tRNA: transfer RNA; rRNA: ribosomal RNA.

**Table 2:** List of genes found in the chloroplast genome of *S. brachyotus*.

Category of genes	Group of genes	Names of genes
Self-replication	Large subunit of ribosome (LSU)	rpl33, rpl20, rpl36, rpl14, rpl16^∗^, rpl22, rpl2(2)^∗^, rpl23(2), rpl32
	Small subunit of ribosome (SSU)	rps16^∗^, rps2, rps14, rps4, rps18, rps12(2)^∗^, rps11, rps8, rps3, rps19, rps7(2), rps15
	RNA polymerase subunits	rpoB, rpoC1^∗^, rpoC2, rpoA
	Ribosomal RNA genes	rrn16(2), rrn23(2), rrn4.5(2), rrn5(2)
	Transfer RNAs (tRNAs)	trnH-GUG, trnK-UUU^∗^, trnQ-UUG, trnS-GCU, trnC-GCA, trnD-GUC, trnY-GUA, trnE-UUC, trnR-UCU, trnG-UCC^∗^, trnT-GGU, trnS-UGA, trnG-GCC, trnfM-CAU, trnS-GGA, trnT-UGU, trnL-UAA^∗^, trnF-GAA, trnV-UAC^∗^, trnM-CAU, trnW-CCA, trnP-UGG, trnI-CAU(2), trnL-CAA(2), trnV-GAC(2), trnI-GAU(2)^∗^, trnA-UGC(2)^∗^, trnR-ACG(2), trnN-GUU(2), trnL-UAG

Photosynthesis	Photosystem I	psaB, psaA, ycf3^∗∗^, psaI, ycf4, psaJ, psaC
	Photosystem II	psbA, psbK, psbI, psbM, psbD, psbC, psbZ, psbJ, psbF, psbE, psbB, psbT, psbN, psbH
	Subunits of NADH dehydrogenase	ndhJ, ndhK, ndhC, ndhB(2)^∗^, ndhF, ndhD, ndhE, ndhG, ndhI, ndhA^∗^, ndhH
	Cytochrome b/f complex	petN, petA, petL, petG, petB^∗^, petD^∗^
	ATP synthase	atpI, atpH, atpF^∗^, atpA, atpE, atpB
	Large chain of rubisco	rbcL

Other genes	Translation initiation factor	infA
	Maturase	matK
	Protease	clpP^∗∗^
	Envelope membrane protein	cemA
	Subunit of acetyl-CoA-carboxylase	accD
	Cytochrome c biogenesis protein	ccsA
	Hypothetical chloroplast reading frames	ycf2(2), ycf15(2), ycf1(2)

^∗^Genes containing a single intron. ^∗∗^Genes containing 2 introns.

## Data Availability

The data that support the findings of this study are openly available in GenBank of NCBI at https://www.ncbi.nlm.nih.gov/, and the accession numbers are provided in Table S1 in Supplementary Materials.

## Supplementary Materials

Supplementary Materials Table S1: list of chloroplast sequences included in the phylogenetic analyses. Figure S1: pictures of *Sonchus brachyotus*.
