# Supplementary material for: Complete Chloroplast Genome Sequence of Sonchus brachyotus Helps to Elucidate Evolutionary Relationships with Related Species of Asteraceae
Source: Biomed Res Int. 2021 Dec 1;2021:9410496. doi: 10.1155/2021/9410496 (PMC8654571; doi:10.1155/2021/9410496)
Supplement: Supplementary Materials — Table S1: list of chloroplast sequences included in the phylogenetic analyses. Figure S1: pictures of Sonchus brachyotus. [file 9410496.f1.zip › concise description of Figure S1.docx]

**Figure S1.** Pictures of *Sonchus brachyotus* transplanted from the shore of the Yellow River in the Anning district of Lanzhou, Gansu Province, China (36°5’10’N, 103°34’47’E), to pots in a laboratory. **A.** plant; B. leaf; **C**. stem; D. flower.
