# Supplementary material for: Complete Chloroplast Genome Sequence of Sonchus brachyotus Helps to Elucidate Evolutionary Relationships with Related Species of Asteraceae
Source: Biomed Res Int. 2021 Dec 1;2021:9410496. doi: 10.1155/2021/9410496 (PMC8654571; doi:10.1155/2021/9410496)
Supplement: Supplementary Materials — Table S1: list of chloroplast sequences included in the phylogenetic analyses. Figure S1: pictures of Sonchus brachyotus. [file 9410496.f1.zip › Table S1(revision).docx]

Table S1 List of chloroplast genome sequences included in the phylogenetic analyses.

| No. | Tribe | Genus name | Species name | Accession number |
| --- | --- | --- | --- | --- |
| 1 | Lactuceae | *Sonchus* | *Sonchus webbii* | NC_042383.1 |
| 2 |  |  | *Sonchus acaulis* | NC_042382.1 |
| 3 |  |  | *Sonchus canariensis* | NC_042381.1 |
| 4 |  |  | *Sonchus boulosii* | NC_042244.1 |
| 5 |  |  | *Sonchus oleraceus* | MG_878405.1 |
| 6 |  |  | *Sonchus arvensis* | NC_054161.1 |
| 7 |  | *Lactuca* | *Lactuca sativa* | NC_007578.1 |
| 8 |  |  | *Lactuca sativa* | DQ383816.1 |
| 9 |  | *Mulgedium* | *Mulgedium tataricum* L. DC | MT_845217.1 |
| 10 |  | *Taraxacum* | *Taraxacum* sp. RHS-2016 | KX499523.1 |
| 11 |  |  | *Taraxacum amplum* | NC_031816.1 |
| 12 |  |  | *Taraxacum obtusifrons* | NC_031815.1 |
| 13 |  |  | *Taraxacum mongolicum* | NC_031396.1 |
| 14 |  |  | *Taraxacum platycarpum* | KU736960.1 |
| 15 |  |  | *Taraxacum officinale* | NC_030772.1 |
| 16 |  |  | *Taraxacum kok-saghyz* | NC_032057.1 |
| 17 |  |  | *Taraxacum brevicorniculatum* | NC_032056.1 |
| 18 | Cynareae | *Cirsium* | *Cirsium japonicum* var. *maackii* | MF034024.1 |
| 19 |  |  | *Cirsium vulgare* | NC_036967.1 |
| 20 |  |  | *Cirsium eriophorum* | NC_036966.1 |
| 21 |  |  | *Cirsium arvense* | NC_036965.1 |
| 22 |  | *Atractylodes* | *Atractylodes macrocephala* | MF034020.1 |
| 23 |  |  | *Atractylodes lancea* | NC_037483.1 |
| 24 |  |  | *Atractylodes chinensis* | NC_037484.1 |
| 25 |  | *Saussurea* | *Saussurea involucrata* | NC_029465.1 |
| 26 |  |  | *Saussurea polylepis* | NC_036490.1 |
| 27 |  |  | *Saussurea chabyoungsanica* | NC_036677.1 |
| 28 |  | *Carthamus* | *Carthamus tinctorius* | NC_030783.1 |
| 29 | Anthemideae | *Chrysanthemum* | *Chrysanthemum indicum* | NC_020320.1 |
| 30 |  |  | *Chrysanthemum* x *morifolium* | NC_020092.1 |
| 31 |  |  | *Chrysanthemum zawadskii* var. *latilobum* | MF034026.1 |
| 32 |  | *Artemisia* | *Artemisia argyi* | NC_030785.1 |
| 33 |  |  | *Artemisia capillaris* | NC_031400.1 |
| 34 |  |  | *Artemisia annua* | MF623173.1 |
| 35 | inuleae | *Leontopodium* | *Leontopodium leiolepis* | NC_027835.1 |
| 36 |  | *Anaphalis* | *Anaphalis sinica* | NC_034648.1 |
| 37 | Astereae | *Aster* | *Aster altaicus* | NC_034996.1 |
| 38 |  |  | *Aster indicus* | NC_040126.1 |
| 39 |  |  | *Aster spathulifolius* | NC_027434.1 |
| 40 | Heliantheae | *Helianthus* | *Helianthus annuus* | NC_007977.1 |
| 41 |  |  | *Helianthus tuberosus* | MG696658.1 |
| 42 |  |  | *Helianthus argophyllus* | NC_030275.1 |
