# Supplementary figures and images for: Complete Chloroplast Genome Sequence of Sonchus brachyotus Helps to Elucidate Evolutionary Relationships with Related Species of Asteraceae
Source: Biomed Res Int. 2021 Dec 1;2021:9410496. doi: 10.1155/2021/9410496 (PMC8654571; doi:10.1155/2021/9410496)

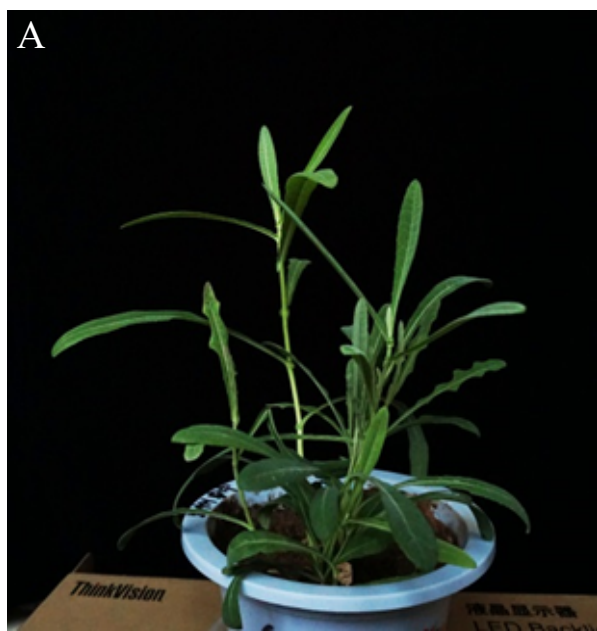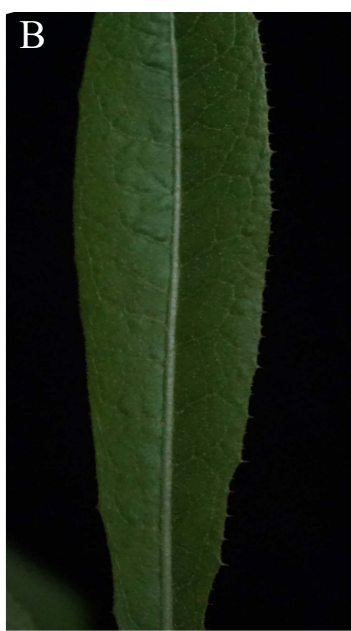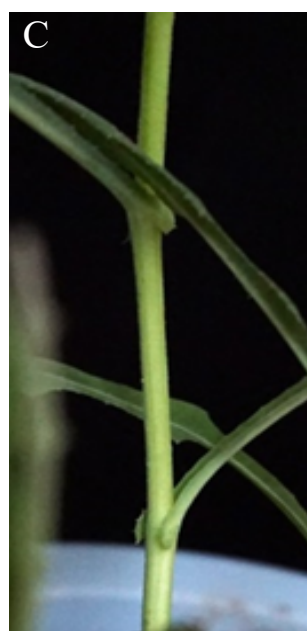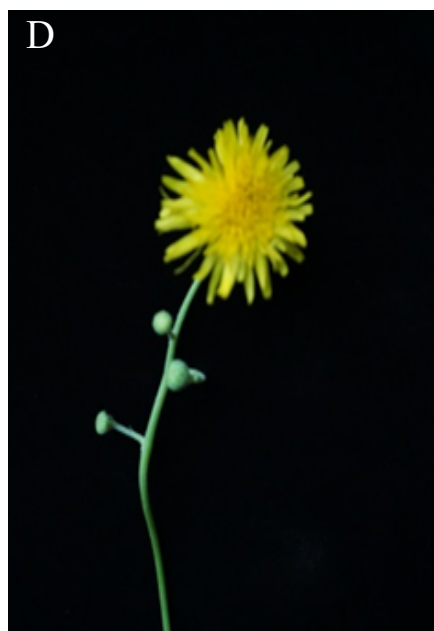

Supplement: Supplementary Materials — Table S1: list of chloroplast sequences included in the phylogenetic analyses. Figure S1: pictures of Sonchus brachyotus. [file 9410496.f1.zip › Fig S1.pdf]
